# Pica (Allotriophagy): An Underestimated Risk Factor for Severe Leptospirosis (Weil’s Diseases)? Report of a Leptospira Septic Shock Successfully Managed with ECMO

**DOI:** 10.3390/idr13030058

**Published:** 2021-07-05

**Authors:** Adam Fabiani, Eugenia Dal Bo, Stefano Di Bella, Marco Gabrielli, Alessandro Bologna, Umberto Albert, Gianfranco Sanson

**Affiliations:** 1 Cardiothoracic-Vascular Department, Azienda Sanitaria Universitaria Integrata, Cattinara University Hospital, 34148 Trieste, Italy; adam.fabiani@asugi.sanita.fvg.it (A.F.); eugenia.dalbo@asugi.sanita.fvg.it (E.D.B.); marco.gabrielli@asugi.sanita.fvg.it (M.G.); alessandro.bologna@asugi.sanita.fvg.it (A.B.); 2 Clinical Department of Medical, Surgical and Health Sciences, University of Trieste, Cattinara University Hospital, 34148 Trieste, Italy; sdibella@units.it (S.D.B.); ualbert@units.it (U.A.); 3 UCO Clinica Psichiatrica, Department of Mental Health, 34128 Trieste, Italy

**Keywords:** acute respiratory distress syndrome, extracorporeal membrane oxygenation, hemorrhagic pneumonia, leptospirosis, pica

## Abstract

Leptospirosis is a zoonosis caused by infection with pathogenic strains of the bacterium Leptospira. The disease can be complicated by pulmonary hemorrhages and acute respiratory distress syndrome, with the mortality rate increasing to 51–100%. We report the case of a 37-year-old man who was admitted to the emergency department with a 6-day history of fever, weakness, vomiting and diarrhea, followed by jaundice. On admission, he presented leukocytosis, thrombocytopenia and acute liver and kidney injuries. His clinical course was critical, as it was immediately complicated by sepsis and severe respiratory failure, requiring haemodialysis, mechanical ventilation and broad-spectrum antibiotic therapy. In the following days, a veno-venous extracorporeal membrane oxygenation (VV-ECMO) was started due to a dramatic deterioration in respiratory function; 20 h later, it was switched to veno-arterial ECMO because of refractory cardiogenic shock. Hantavirus or Leptospira infection etiology was suspected, so penicillin G and methylprednisolone were initiated as an empirical therapy and subsequently confirmed after a laboratory diagnosis of leptospirosis. Although the clinical course was further complicated by hemorrhagic pneumonia, a gradual, full recovery occurred, and the patient was discharged from the hospital. After excluding other sources of contact with Leptospira-infected material, an unsuspected abnormal eating behavior was identified as the most probable cause of the patient’s Leptospira infection.

## 1. Introduction

Leptospirosis is a zoonosis caused by pathogenic strains of the bacterium Leptospira [1]. Although spread worldwide, leptospirosis is more representative in tropical and financially poor areas, where transmission is more favorable [2]. A large proportion of cases (48%, 95% CI 40–61%) and deaths (42%, 95% CI 34–53%) occur in adult males aged 20–49 [3]. The most common symptoms of leptospirosis include fevers, headaches and myalgia, sometimes with hemorrhage or encephalitis as the initial presentation [4,5]. However, leptospirosis can be complicated by pulmonary hemorrhages and acute respiratory distress syndrome (ARDS), with the mortality rate increasing up to 100% [6]. Extracorporeal membrane oxygenation (ECMO) can be considered for patients with untreatable respiratory failure from leptospirosis. We describe a particular, biphasic modality of ECMO support in a severe case of leptospirosis acquired via a never previously documented modality of infection, whose diagnosis was complicated by a concomitant suspected intoxication.

## 2. Case Presentation

### 2.1. Time 0: Admission in the Emergency Department

**Figure 1:**
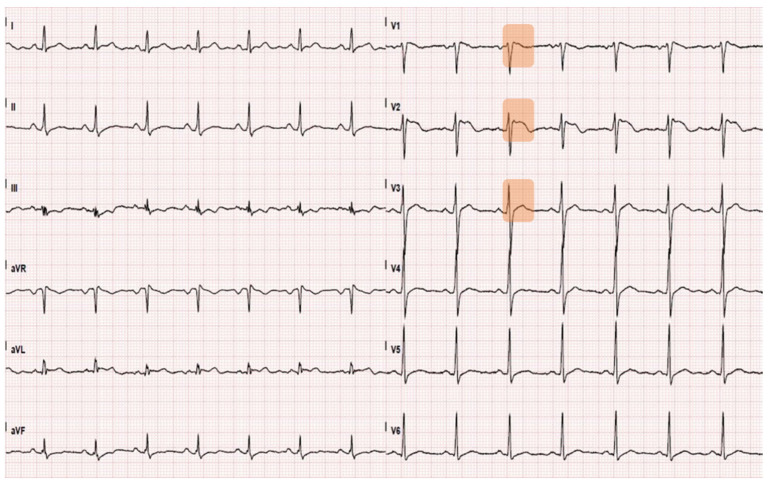
Electrocardiogram on admission showing a Brugada type II pattern (saddleback shaped ST elevation in V1–V3, examples highlighted).

A 37-year-old gentleman was admitted to the emergency department (ED) of an Italian University Hospital due to the onset of jaundice after six days of fever, diarrhea, vomiting and weakness. The patient had a solitary kidney secondary to a post-traumatic nephrectomy. He did not take any medication and had no allergies. Upon hospital admission, he was alert and claimed to experience dizziness. As a sole relevant anamnestic information, the patient reported the ingestion of a few *Taxus baccata* berries a week earlier; however, the patient declared having eaten only the non-toxic red arils of the berries and no symptom of severe poisoning (e.g., cardiac arrhythmias, shock) was present at the time. The patient had normal vital signs. Neither a physical examination nor an ultrasound scan found critical issues. No skin rash was observed. An electrocardiogram (ECG) showed a normal sinus rhythm with a Brugada type II pattern (Figure 1).

Blood tests showed cholestatic hepatitis, a severe kidney injury and elevated creatine phosphokinase (CK) consistent with rhabdomyolysis. Urine (benzodiazepines, cocaine, opiates, cannabinoids, barbiturates, methadone, amphetamines) and blood (alcohol) toxicology tests were negative. Blood cultures (BD Bactec^®^) and viral hepatitis (HAV, HBV, HCV, HEV) serology were requested (IgM and IgG for HAV and HEV; total antibodies for HCV and HBsAg, HBsAb, HBcAb, HbeAg, HBeAb for HBV), and continuous renal replacement therapy, plus cytokine adsorber (Cytosorb^®^), was started. After 24 h from ED admission, his clinical conditions started worsening, with hemodynamic instability and severe respiratory failure, requiring invasive mechanical ventilation (MV) and intensive care unit (ICU) admission.

### 2.2. 12 h from ED Admission: Transfer to the General Intensive Care Unit

**Figure 2:**
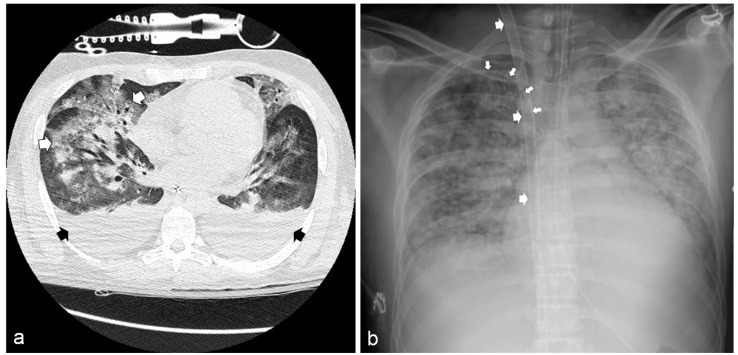
(**a**) Chest computed tomography showing bilateral pleural effusion (black arrows) and diffuse ground-glass infiltrates (white arrows). (**b**) Chest radiography showing the bi-caval dual-lumen catheter placed in the right jugular vein (large arrows) and the central venous catheter in the right subclavian vein (small arrows).

Chest X-ray and chest computed tomography (CT) scans showed bilateral pneumonia with ground-glass infiltrates (Figure 2a). Clinical and laboratory findings were consistent with the diagnosis of septic shock with multi-organ failure (MOF). Hemodynamic stability was restored through high-dose norepinephrine (up to 1.9 μg/kg/min). A broad-spectrum antibiotic therapy was also started (ceftazidime 4 g/day and meropenem 2 g/day). Nevertheless, a progressive, severe deterioration of respiratory functions was observed. Thirty-six hours after ED admission, the partial pressure of oxygen to fraction of inspired oxygen ratio (PaO_2_/FiO_2_) fell to 51.5, despite volume-controlled ventilation (FiO_2_ = 1; PEEP = 7 cm H_2_O; respiratory rate = 20 breaths/min; tidal volume = 500 mL); therefore, the ECMO team was immediately alerted, and the patient was admitted to the cardiac surgery ICU.

### 2.3. 36 h from ED Admission: Transfer to the Cardiac Surgery Intensive Care Unit

After placing an Avalon Elite^®^ Bi-Caval Dual-Lumen Catheter through the right internal jugular vein under trans-esophageal ultrasound guidance, a veno-venous ECMO (VV-ECMO) was started. A further triluminal central catheter was positioned through the right subclavian vein for hemodynamic and oxyphoretic monitoring and drug administration (Figure 2b).

During the following 20 h, the patient experienced a good tolerance to VV-ECMO. Unfortunately, after this period, a cardiogenic shock due to a severe left ventricular dysfunction (ejection fraction 20%) occurred, which was exacerbated by the contemporary onset of sustained supraventricular tachycardia. A sinus rhythm was restored after delivering six 150 Joule cardioversion shocks and administering intravenous antiarrhythmic drugs (amiodarone 150 mg; metoprolol 4 mg; diltiazem 25 mg). Since hemodynamics and tissue perfusion did not improve his condition, despite the use of high-dose norepinephrine, an emergency switch from VV- to veno-arterial ECMO (VA-ECMO) was promptly established: the Avalon cannula was used exclusively to draw the venous blood, which, after being oxygenated via ECMO, was reinfused through a newly positioned right femoral arterial cannula. A transient ischemia of the cannulated lower limb subsequently appeared, which was resolved by positioning an additional cannula for distal perfusion. During this time, sedation, MV, cytokine adsorption and renal replacement were continued. The patient also experienced a transient third cranial nerve (oculomotor) palsy; however, his laboratory blood parameters progressively improved.

The hepatitis serology and blood culture results were negative. Given the persistent sepsis with pulmonary and hepatorenal presentation, either Hantavirus or Leptospira infection etiology was suspected, and blood samples were sent to the laboratory for appropriate serology and molecular biology testing. Meanwhile, penicillin G (12 million units/day in continuous infusion, renal dosing) and intravenous methylprednisolone were initiated as empirical therapy (starting with 40 mg/day, then gradually decremented and discontinued after 7 days). At the same time, cytokine adsorption was discontinued to avoid the risk of unwanted antibiotic clearance. Hantavirus serology and blood polymerase chain reaction (PCR) were negative, while PCR and IgM (ELISA) were positive for Leptospira (an *interrogans* species was subsequently identified), so Weil’s syndrome was diagnosed and both antibiotic and steroid therapies were confirmed and continued for 10 and 6 days, respectively. On day six, the patient experienced a slow but progressive clinical improvement: vasopressors were progressively reduced until the point of stopping, diuresis resumed and dialysis was ceased. ECMO was discontinued, and the patient was transferred back to the general ICU on day 10.

### 2.4. 10 Days from ED Admission: Transfer to the General Intensive Care Unit

A new exploration of the patient’s lifestyle was performed by interviewing his parents to understand how the infection was contracted. The patient was actually unemployed and had no other at-risk activity (e.g., farming). In the last few months, he showed abnormal behavior, and began eating strange food, such as wild berries or the raw meat of wildlife animals (e.g., lizards), and drinking stagnant water. He had no prior diagnosed mental disorder nor had he ever received treatment for a psychiatric condition; more specifically, he never showed any signs or symptoms of autism spectrum disorder or another neurodevelopmental disorder.

On day 13, the patient was extubated. However, MV was later restarted because of a new severe respiratory failure. A further chest CT scan revealed severe hemorrhagic pneumonia, compatible with an immune-mediated process secondary to leptospirosis. Intravenous steroid therapy was restarted at higher doses (methylprednisolone 80 mg/day) to contrast the likely immune-mediated damage pathogenesis, and progressively tapered for a 7-day cycle. Over the next three days, a gradual recovery occurred until the definitive restoration of spontaneous breathing and extubation.

Vital, blood, respiratory and ECMO parameters describing the most relevant steps of the patient’s clinical course are reported in Table 1 and Table 2.

### 2.5. 16 Days from ED Admission: Transfer to the Cardiology Department

After a 6-day stay in the general ICU, the patient was transferred to the cardiology ICU for further cardiocirculatory monitoring and assessment of the heart disease. The patient confirmed the abnormal eating behavior previously referred by his parents, explaining it as the desire to try new experiences. Since no other likely source of contact with Leptospira-infected material was highlighted by an in-depth patient interview, this eating behavior was identified as the most plausible source of Leptospira infection.

### 2.6. 33 Days from ED Admission: Discharged from Hospital

The hospital discharge occurred after a total of 33 days. The patient was transferred to a rehabilitation center, where a motor and respiratory recovery program was started. At the follow-up 10 days after hospital discharge, the patient showed a full recovery, and all laboratory blood tests were in the acceptable range.

## 3. Discussion

We presented a case of severe Leptospiral infection. Although not recognized by clinicians, on hospital admission, the patient clearly presented with Weil’s disease features, a severe form of leptospirosis accounting for 5–10% of the cases, which can develop as a unique progressive clinical picture or—as in our case—can be the second, more serious stage of a biphasic disease. Weil’s disease is characterized by a triad of jaundice, renal failure and hemorrhagic diathesis [7]. While in ED, jaundice and renal failure were easily recognized, the patient did not show obvious signs of bleeding; however, platelet count was very low and—despite the normal hemoglobin level—could have suggested the presence of hemorrhagic diathesis. Thrombocytopenia is emblematic of Weil’s disease (up to 50% prevalence), is typically related with renal failure and is associated with a poor prognosis [8]. In addition, the patient showed rhabdomyolysis as an additional known clinical feature that was potentially co-responsible for the kidney injury [9].

Additionally, in a Leptospira infection, neurological complications (e.g., encephalitis, optic neuritis, cranial nerve paresis) may occur [10]. During his clinical course, the patient experienced a transient oculomotor nerve palsy. The heart is probably more commonly involved than reported in the literature, but severe myocarditis and heart failure have seldom been documented, and echocardiographic evidence of myocardial dysfunction has not been adequately demonstrated [11]. In the present case, the patient suffered from severe left ventricular failure and tachyarrhythmia. Moreover, a type II Brugada ECG pattern was observed. The presence of both life-threatening tachyarrhythmias and ECG phenotypes of Brugada syndrome have been observed as being associated with both leptospirosis [11] and yew (*Taxus baccata*) poisoning [12,13]. When related to leptospirosis, cardiac involvement in the form of ECG changes (e.g., non-specific conduction system abnormalities, arrhythmias, alterations similar to acute pericarditis, T-wave inversions, S-T segment elevations), and myocarditis can occur as early as the leptospiremic phase; this is probably secondary to toxin-mediated vascular damage, leading to intimitis and perivascular and subendocardial inflammation [14]. The cardiotoxic effects of yew generally develop 1–3 days after yew ingestion [15], and are primarily related to the presence of taxine alkaloids (mainly taxine B, associated with its calcium channel antagonism and early sodium current inhibition effects), which have a strong toxic effect on the heart by depressing myocardial contractility and increasing atrio-ventricular conduction time, resulting in the widening of QRS complexes, II/III degree atrio-ventricular blocks and, ultimately, cardiac arrest [15,16]. In the reported case, both causes may have played a role in determining the cardiac complications. Although the patient claimed having ingested only non-toxic parts of the *Taxus baccata* plant, the possible psychiatric problems highlighted later could raise some doubts. However, the time interval between the yew berries’ ingestion and the onset of such severe cardiovascular complications (7–8 days) makes the contribution of poisoning less likely.

An even more serious form of Leptospiral infection exists, namely the severe pulmonary form of leptospirosis (SPFL), which is characterized by intra-alveolar hemorrhage leading to severe, life-threatening acute respiratory failure. SPFL presents in less than 5% of cases, usually in the early course of the disease [17]. This was the case with our patient, who presented concomitant septic shock, SPFL and heart failure, which led him to a near-death condition. Weil’s disease complicated by SPFL is associated with a mortality rate up to 70% in the absence of prompt and adequate treatment [3]. Notably, in patients with pulmonary involvement, hemodynamic disturbances were associated with even higher mortality rates [8]. In our case, given the quick worsening of respiratory failure, the prompt decision to start VV-ECMO support—shifted to VA-ECMO because of the subsequent hemodynamic deterioration—provided a transition period for respiratory and hemodynamic recovery, and was probably the trump card in determining the patient’s survival with a good neurological outcome.

ECMO support in severe leptospirosis was previously described as an effective treatment for reducing patient mortality. Twelve recent case reports describe the use of a V-V ECMO in patients with leptospirosis complicated by ARDS and hemorrhagic pneumonia; only one patient died [6,18,19,20]. Two case reports described the use of V-A ECMO in two patients affected by complicated leptospirosis with (1) right ventricular overload and myocarditis and (2) cardiogenic shock and pulseless electrical activity, respectively. Both patients survived [21,22]. To the best of our knowledge, this was the first case describing an initial use of VV-ECMO as a pulmonary bypass, followed by a rapid conversion to VA-ECMO when cardiogenic shock occurred.

As soon as the diagnosis of leptospirosis was suspected, the patient was immediately treated with appropriate antibiotics and high doses of intravenous steroids to counteract the important immune-mediated response; however, strong evidence for the use of routine steroids is lacking [18,23].

Precise data about the overall incidence of Leptospira infection in humans in Italy are scarce. A previous study documented an annual incidence of leptospirosis ranging from 0.1 to 13.1 per million inhabitants on a regional basis [24], while a more recent study observed an overall apparent prevalence of 4.26% [25]. Leptospirosis is present and often endemic worldwide, and it is sustained by the persisting colonization of the proximal renal tubules of both wild and domestic carrier mammals, such as mice, rats, dogs, swine, bovines, bats, rodents and marsupials [25]. Infected animals spread Leptospira through their urine, infecting humans either directly or through contaminated soil, objects or water. Accordingly, the infection risk is related to occupational activities (e.g., agricultural activities, mining, sewage working, livestock farming, veterinary medicine), recreational water immersion, poor living conditions and seasonal tropical rainfall [2,8]. Consequently, a singularity of this case report is the source of the infection.

Pica (also named allotriophagy) is one of the feeding and eating disorders classified in the *Diagnostic and Statistical Manual of Mental Disorders*, *Fifth Edition*, and it is characterized by the persistent eating of non-nutritive, non-food substances, such as soil, sand, paper, wood, cotton, etc., over a period of at least one month. In this case, the patient’s parents reported that he used to eat inedible berries and raw meat of wild animals, and used to drink dirty rain water, without showing any signs/symptoms of another mental disorder. Based on all available evidence, he never suffered from a neurodevelopmental disorder or showed signs/symptoms of a manic episode. From an epidemiological point of view, this modality of leptospirosis contagion is very uncommon, and this might the first case to be reported.

## 4. Conclusions

In “Western” countries, leptospirosis is quite uncommon and may lead to life-threatening clinical conditions. This disease should be considered in the differential diagnosis of a patient who presents fever, jaundice, renal failure and haemorrhagic diathesis, possibly associated with mild-to-severe respiratory symptoms. Great attention must always be paid to a patient’s medical history, particularly focusing on a possibly unusual modality of contagions due to a maladaptive eating behavior/disorder when patient presents symptoms compatible with leptospirosis, mostly in geographical areas where leptospirosis is a rare phenomenon. In the present case report, the diagnosis was challenging for clinicians living in a non-endemic area (i.e., not used to include leptospirosis in the differential diagnosis), and the recent assumption of *Taxus baccata* berries acted as a confounding factor in clinical reasoning. However, the epidemiologic, laboratory, microbiology, imaging features and clinical picture were consistent with severe leptospirosis with a biphasic course.

If diagnosis and treatments are promptly and correctly carried out, prognosis can be completely favorable. Intensive care is needed, and ECMO should be considered early in the most severe cases in which pulmonary or cardiac events are involved.

## Figures and Tables

**Table 1:** Evolution of patient’s clinical parameters during the hospital stay.

Chronology	h 0	h 24	h 36	h 55	Day 6	Day 10	Day 16
	ED Admission	ICU Admission	VV-ECMO Start	VA-ECMO Start	VA-ECMO Stop	CS-ICU Discharge	MV Stop
Vital signs							
Blood pressure (mm Hg)	110/70	110/55	99/55	30/15	120/60	115/55	110/65
Heart rate (beats per minute)	87	115	90	150	95	75	72
Body temperature (°C)	36.6	38.4	37.5	37.5	36.5	36.5	36.2
Arterial blood gas							
pH	7.48	7.21	7.33	7.28	7.38	7.40	7.42
PaCO_2_ (mm Hg)	39.1	65.9	41.6	34	42.8	40.1	41.3
PaO_2_ (mm Hg)	62.4	51.5	132	90	132	114	93
PaO_2_/FiO_2_	297	51.5	/	/	/	380	442
SaO_2_ (%)	94.7	83.3	95.7	93.6	96.3	99.5	99.2
HCO_3_^−^ (mmol/L)	29.7	26.5	22.3	18	25.1	24.9	23.9
Lactate (mmol/L)	1.3	2.6	2.7	5.7	0.8	0.8	0.4
Biochemistry							
Aspartate aminotransferase (U/L)	77	61	55	59	285	209	58
Alanine aminotransferase (U/L)	61	45	39	37	117	213	72
Bilirubin (mg/dL)	12.3	12.95	n.a.	8.4	10.3	6.4	1.59
Creatinine (mg/dL)	9.2	4.16	4.28	3.80	1.36	2.03	1.18
Urea (mg/dL)	177	59	64	73	57	71	54
Hemoglobin (g/dL)	13.7	15.1	11.8	10.5	8.3	10.4	11.6
White-cell count (per mm^3^)	18,700	19,710	25,890	54,760	33,320	16,610	11,164
Platelet count (per mm^3^)	30,000	74,000	85,000	123,000	32,000	130,000	337,000
Creatine phosphokinase (U/L)	1309	503	310	241	19,043	4053	1254
High sensitivity troponin I (ng/L)	911	242	198	299	149	34	28
C-reactive protein (mg/L)	187.5	188.7	253	392	46	91.3	117

ED: emergency department. VV-ECMO: veno-venous extracorporeal membrane oxygenation. VA-ECMO: veno-arterial extracorporeal membrane oxygenation. ICU: intensive care unit. CS-ICU: cardiac surgery ICU.

**Table 2:** Ventilation and ECMO parameters during the hospital stay.

Chronology	h 0	h 24	h 36	h 55	Day 6	Day 10	Day 16
	ED Admission	ICU Admission	VV-ECMO Start	VA-ECMO Start	VA-ECMO Stop	CS-ICU Discharge	MV Stop
Ventilation parameters							
Modality	SB	MV-CV	MV-PSV	MV-SIMV	MV-PSV	MV-PSV	SB
FiO_2_ (%)	21	100	40	40	40	30	21
Respiratory rate	/	20	/	14	/	/	15
Tidal volume (mL)	/	500	/	440	/	/	/
PEEP (cm H_2_O)	/	7	12	10	8	8	/
Support press. (cm H_2_O)	/	/	12	/	10	10	/
ECMO parameter							
Blood flow (rpm; L/min)	/	/	3400; 3.5	3050; 4.7	1950; 1.5	/	/
Gas flow (FiO_2_; L/min)	/	/	1.0; 3.0	0.7; 5.5	0.7; 1.0	/	/

ED: emergency department. VV-ECMO: veno-venous extracorporeal membrane oxygenation. VA-ECMO: veno-arterial extracorporeal membrane oxygenation. ICU: intensive care unit. CS-ICU: cardiac surgery ICU. SB: spontaneous breathing. MV: mechanical ventilation. CV: controlled volume. PSV: pressure support ventilation. SIMV: synchronized intermittent mandatory ventilation. rpm: revolutions per minute.

## Data Availability

Not applicable.
